# Weight-reduction through a low-fat diet causes differential expression of circulating microRNAs in obese C57BL/6 mice

**DOI:** 10.1186/s12864-015-1896-3

**Published:** 2015-09-16

**Authors:** Ching-Hua Hsieh, Cheng-Shyuan Rau, Shao-Chun Wu, Johnson Chia-Shen Yang, Yi-Chan Wu, Tsu-Hsiang Lu, Siou-Ling Tzeng, Chia-Jung Wu, Chia-Wei Lin

**Affiliations:** Department of Plastic and Reconstructive Surgery, Kaohsiung Chang Gung Memorial Hospital and Chang Gung University College of Medicine, No. 123, Ta-Pei Road, Niao-Song District, Kaohsiung City, 833 Taiwan; Department of Neurosurgery, Kaohsiung Chang Gung Memorial Hospital and Chang Gung University College of Medicine, No. 123, Ta-Pei Road, Niao-Song District, Kaohsiung City, 833 Taiwan; Department of Anesthesiology, Kaohsiung Chang Gung Memorial Hospital and Chang Gung University College of Medicine, No. 123, Ta-Pei Road, Niao-Song District, Kaohsiung City, 833 Taiwan

**Keywords:** Diet-induced Obesity, MicroRNAs, High-fat Diet, Low-fat Diet

## Abstract

**Background:**

To examine the circulating microRNA (miRNA) expression profile in a mouse model of diet-induced obesity (DIO) with subsequent weight reduction achieved via low-fat diet (LFD) feeding.

**Results:**

Eighteen C57BL/6NCrl male mice were divided into three subgroups: (1) control, mice were fed a standard AIN-76A (fat: 11.5 kcal %) diet for 12 weeks; (2) DIO, mice were fed a 58 kcal % high-fat diet (HFD) for 12 weeks; and (3) DIO + LFD, mice were fed a HFD for 8 weeks to induce obesity and then switched to a 10.5 kcal % LFD for 4 weeks. A switch to LFD feeding led to decreases in body weight, adiposity, and blood glucose levels in DIO mice. Microarray analysis of miRNA using The Mouse & Rat miRNA OneArray® v4 system revealed significant alterations in the expression of miRNAs in DIO and DIO + LFD mice. Notably, 23 circulating miRNAs (mmu-miR-16, mmu-let-7i, mmu-miR-26a, mmu-miR-17, mmu-miR-107, mmu-miR-195, mmu-miR-20a, mmu-miR-25, mmu-miR-15b, mmu-miR-15a, mmu-let-7b, mmu-let-7a, mmu-let-7c, mmu-miR-103, mmu-let-7f, mmu-miR-106a, mmu-miR-106b, mmu-miR-93, mmu-miR-23b, mmu-miR-21, mmu-miR-30b, mmu-miR-221, and mmu-miR-19b) were significantly downregulated in DIO mice but upregulated in DIO + LFD mice. Target prediction and function annotation of associated genes revealed that these genes were predominantly involved in metabolic, insulin signaling, and adipocytokine signaling pathways that directly link the pathophysiological changes associated with obesity and weight reduction.

**Conclusions:**

These results imply that obesity-related reductions in the expression of circulating miRNAs could be reversed through changes in metabolism associated with weight reduction achieved through LFD feeding.

**Electronic supplementary material:**

The online version of this article (doi:10.1186/s12864-015-1896-3) contains supplementary material, which is available to authorized users.

## Background

Obesity is associated with insulin resistance and an abnormal inflammatory response [1], and the strong associations suggest that adipose tissue plays a prominent role in the onset and progression of these comorbidities [2]. White adipose tissue (WAT) has been characterized as an endocrine organ [3], as it produces endocrine-acting peptides such as leptin, and it is metabolically important, with excess levels being associated with metabolic syndrome [4, 5]. High fat uptake leads to metabolic alterations in adipose tissue that increase the levels of circulating free fatty acids in the blood [6]. This leads to macrophage activation and the production of proinflammatory cytokines via Toll-like receptors, resulting in inflammation in adipose tissue [6]. When allowed *ad libitum* access to a high-fat diet (HFD), C57BL/6J mice develop insulin resistance and obesity in a manner that resembles disease progression in humans [7]. Increased energy expenditure and decreased energy intake are the two most commonly recommended lifestyle changes to reduce adiposity and restore insulin sensitivity in the treatment of diet-induced obesity (DIO) and associated comorbidities [8]. Calorie restriction is effective in improving insulin sensitivity and decreasing both body weight and percent body fat [9]. In addition, reductions in body weight and improvements in insulin sensitivity can also be achieved by reducing the percentage fat in a diet, i.e., by switching from a HFD to a low-fat diet (LFD) [10].

MicroRNAs (miRNAs) are endogenous small RNAs that post-transcriptionally regulate gene expression, and they have been demonstrated to have important roles in numerous disease processes. There is growing evidence that miRNAs play an important role in regulating adipose tissue pathways that control a range of processes, including adipogenesis, insulin resistance, and inflammation [11–13]. Many miRNAs are dysregulated in the metabolic tissues of obese animals and humans, potentially contributing to the pathogenesis of obesity-associated complications [11–13]. In addition, recent studies identified several miRNAs expressed in metabolic organs that could be used as feasible therapeutic targets for obesity and its consequent pathologies [11, 13]. Recently, circulating serum miRNAs were found to display specific expression patterns, suggesting that miRNA profiles may represent fingerprints for various diseases [14, 15]. In addition, despite the ubiquitous presence of ribonucleases, serum miRNAs levels are remarkably stable and reproducible [16, 17], and they function in cell-to-cell communication [18]. Currently, how changes in miRNA profiles might affect adipose tissue at the functional and molecular level and to what extent they differ in response to weight-reduction strategies are not well understood. This information is important in the development of dietary anti-obesity interventions [19]. As circulating miRNAs potentially play an important role in regulating the pathophysiology of obesity and they are potential therapeutic targets, we hypothesized the weight reduction may change the circulating miRNAs expression. Our study aim was to profile the expression of circulating miRNAs in a mouse model of DIO with subsequent weight reduction achieved through LFD feeding.

## Methods

### Ethics statement

This study was conducted in strict accordance with guidelines on the use of laboratory animals, and every effort was made to minimize the suffering of affected animals. Animal protocols were approved by the IACUC of Chang Gung Memorial Hospital, Taiwan (permission number No. 2012091002).

### Animal experiments

C57BL/6NCrl mice were purchased from BioLasco (Taipei, Taiwan). Animals were housed, and surgical procedures, including analgesia, were performed in an Association for Assessment and Accreditation of Laboratory Animal Care International-accredited SPF facility according to national and institutional guidelines. In this experiment, 18 male, wild-type C57BL/6NCrl mice were randomly assigned to three subgroups (*n* = 6 in each group) as follows: (1) control, mice were fed a standard AIN-76A (fat: 11.5 kcal %) diet *ad libitum* for 12 weeks; (2) DIO, mice were fed a 58 kcal % HFD (D12331; Research Diets Inc., New Brunswick, NJ) *ad libitum* for 12 weeks to induce obesity; and (3) DIO + LFD, mice were fed a 58 kcal% HFD (D12331) *ad libitum* for 8 weeks to induce obesity and then fed a 10.5 kcal% LFD (D 12329; Research Diets Inc.) for 4 weeks. Weight measurements were performed weekly, and a glucose tolerance test was performed at the beginning and end of the experiment to confirm that HFD-fed mice developed an obese and glucose intolerance phenotype. Briefly, mice were fasted for 5 h, and baseline blood glucose levels were measured with an Accu-Check Advantage blood glucose meter (Roche, New Jersey, USA) using blood collected from the tail vein. Mice (*n* = 6 in each group) were injected intraperitoneally with 2 g of glucose per kilogram body weight in sterile PBS. The glucose level was measured via tail vein blood (~10 μL) at t = -30 and 0 (pre) and t = 15, 30, 60, 90, and 120 min after the glucose infusion. Data were averaged and graphed as blood glucose level as a function of time. To reflect the circulating levels of glucose during the glucose tolerance test (GTT), we calculated the total area under the curve (AUC) of the glucose concentration versus time by the linear trapezoidal rule for the period of 0 - 120 min after glucose infusion. To avoid the effect of loss of blood in GTT experiment or uncertain effect or repeat tail punctures on subsequent miRNAs expression and cytokine assay, additional groups of mice under the same model were used for further experiments. After the end of the experiment, all mice were euthanized, and the abdominal mesenteric WAT of each mouse was removed and weighed. The adipose tissue block embedded in paraffin was sectioned at 5 μm to measure the adipocyte area. Three 5 μm-thickness sections of the same fat specimen at 50 μm distance was mounted on glass plate and stained with hematoxylin and eosin. Two different microscopic fields (magnification × 100) per plate were photographed and 100 adipose cells were arbitrarily selected in the center of field and their cell diameters were assessed by tracing the outline of each adipocyte. The mean adipocyte area was measured from the WAT of control and experimental mice (*n* = 4 in each group) using Image-Pro Plus image analysis software (Carl Zeiss, Oberkochen, Germany) and expressed in terms of square micrometers. The cells were randomly chosen, and the person analyzing the images was blinded to the group assignments. At the indicated time of the experiment, 1 mL of whole blood was collected via cardiac puncture into a plain tube and allowed to clot for 1 h. Samples were centrifuged at 3000 × *g* for 10 min, and sera were aliquoted and stored at −80°C until further analysis.

### Cytokine assays

Serum cytokine concentrations were analyzed using two complementary Bio-Plex suspension arrays (M60-00003J7 and M60-00007NY) covering all cytokine biomarkers potentially involved in inflammation (*n* = 6 in each group). Eleven biomarkers (IL-1β, IL-2, IL-4, IL-5, IL-6, IL-10, IL-12(p70), IL-17, GM-CSF, IFN-γ, and TNF-α) were assessed simultaneously using the Bio-Plex system (BioRad, Hercules, CA). Assays were performed on four biological replicates per the manufacturer’s instructions. Results are expressed in picograms per milliliter of serum.

### RNA isolation and preparation

Total RNA was extracted from serum using the mirVana™ miRNA Isolation Kit (Life Technologies, NY). Purified RNA was quantitatively evaluated by measuring its absorbance at 260 nm using an SSP-3000 Nanodrop spectrophotometer (Infinigen Biotechnology, Inc., City of Industry, CA), and RNA quality was assessed using a Bioanalyzer 2100 (Agilent Technologies, Santa Clara, CA). Total RNA (10 ng) was reverse-transcribed into cDNA using a TaqMan miRNA Reverse Transcription Kit (Applied Biosystems, Foster City, CA). Target miRNAs were reverse-transcribed using sequence-specific stem-loop primers, and cDNA was used for quantitative real-time polymerase chain reaction (qPCR).

### miRNA microarray analysis

The Mouse & Rat miRNA OneArray® v4 (Phalanx Biotech Group, Hsinchu, Taiwan) array used in this experiment contains 144 experimental control probes, 1157 unique mouse miRNA probes, and 680 rat miRNA probes, based on the miRBase 18 database. Three biological replicates of each group of mice were used in miRNA microarray experiments. Mouse genome-wide miRNA microarray experimental and statistical analyses were performed by Phalanx Biotech Group. Briefly, fluorescent targets were prepared from 2.5 μg of total RNA using the miRNA ULS™ Labeling Kit (Kreatech Diagnostics, Amsterdam, Netherlands). Labeled miRNA targets enriched using NanoSep 100K (Pall Corporation, Port Washington, NY) were hybridized to The Mouse & Rat miRNA OneArray® v4 in Phalanx hybridization buffer in the OneArray® Hybridization Chamber. After overnight hybridization at 37 °C, non-specifically bound targets were removed by three washing steps (Wash I, 37 °C, 5 min; Wash II, 37 °C, 5 min and 25 °C, 5 min; and Wash III, rinse 20 times). Slides were dried by centrifugation and scanned using an Axon 4000B scanner (Molecular Devices, Sunnyvale, CA). The signal intensities of Cy5 fluorescence in each spot were analyzed using GenePix 4.1 software (Molecular Devices, Sunnyvale, CA) and processed using R language (http://www.r-project.org/) with two packages: limma (http://www.bioconductor.org/packages/release/bioc/html/limma.html) and genefilter (http://www.bioconductor.org/packages/release/bioc/html/genefilter.html). Spots with flag < 0 were filtered out, and the remaining spots were log 2 transformed and normalized using the 75 % media scaling normalization method. Normalized spot intensities were converted into gene expression ratios between the control and treatment groups. Spots with expression ratios ≤0.5 or ≥2, as well as with *p* < 0.05, were selected for further analysis. Differentially expressed miRNAs were subjected to hierarchical cluster analysis using average linkage and Pearson’s correlation as the measure of similarity. The miRNA array data have been deposited in the NCBI Gene Expression Omnibus with the accession number GSE61005. Five miRNAs detected by array analysis were selected and quantified by qPCR using the Applied Biosystems 7500 Real*-*Time PCR System (Life Technologies) to confirm the upregulation of miRNA expression in the DIO + LFD group. Twenty-five femtomoles of single-stranded cel-miR-39 synthesized by Invitrogen (Carlsbad, CA) was spiked into 400 μL of serum as an internal control for the expression of each miRNA.

### Target prediction, GO enrichment, and KEGG pathway analyses

Target prediction was performed to identify the target genes of the identified dysregulated miRNAs by integrating all three public databases (TargetScan, PicTar, and miRanda). This method firstly mapped all target gene candidates to GO terms in the database (http://www.geneontology.org/), calculated gene numbers for each term, and then used a hypergeometric test to find significantly enriched GO terms in target gene candidates compared to the reference gene background. Bonferroni’s correction for the *p*-value was used to obtain a corrected *p*-value. GO terms with corrected *p*-values ≤ 0.05 were defined as significantly enriched in target gene candidates. To reveal the main pathways in which the target gene candidates are involved, pathway analysis using a major public pathway-related database, KEGG, was performed to identify significantly enriched metabolic pathways or signal transduction pathways in target gene candidates compared with the whole reference gene background. Genes with FDR ≤ 0.05 were considered significantly enriched among the target gene candidates.

### Statistical analysis

The body weight of mice are expressed as the mean with a confidence interval. All other experimental data are expressed as the mean ± standard error of the mean. Analysis of variance combined with a Bonferroni post hoc correction was performed to identify significant differences in body weight, weight of fat, adipocyte area, glucose levels, and serum cytokine levels. A *p* value of 0.05 was regarded as the level of statistical significance.

## Results

### LFD decreased body weight and adiposity

**Fig. 1:**
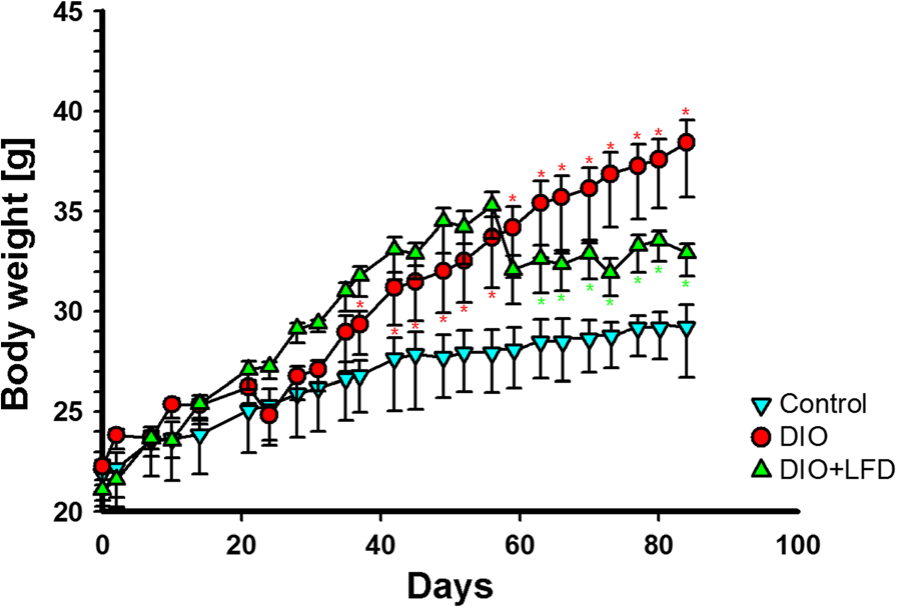
The body weight of C57BL/6NCrl mice on a weekly basis till 12 weeks (*n* = 6 in each group). Control: the mice fed with the standard diet; DIO: the mice fed with high-fat diet; DIO + LFD: the mice fed with high-fat diet for 8 weeks to induce obesity, then change to low-fat diet for subsequent 4 weeks. *(red color), *P* < 0.05 vs. control. *(green color), *P* < 0.05 vs. DIO. The body weight of mice are expressed as the mean a ± 80 % confidence interval

**Fig. 2:**
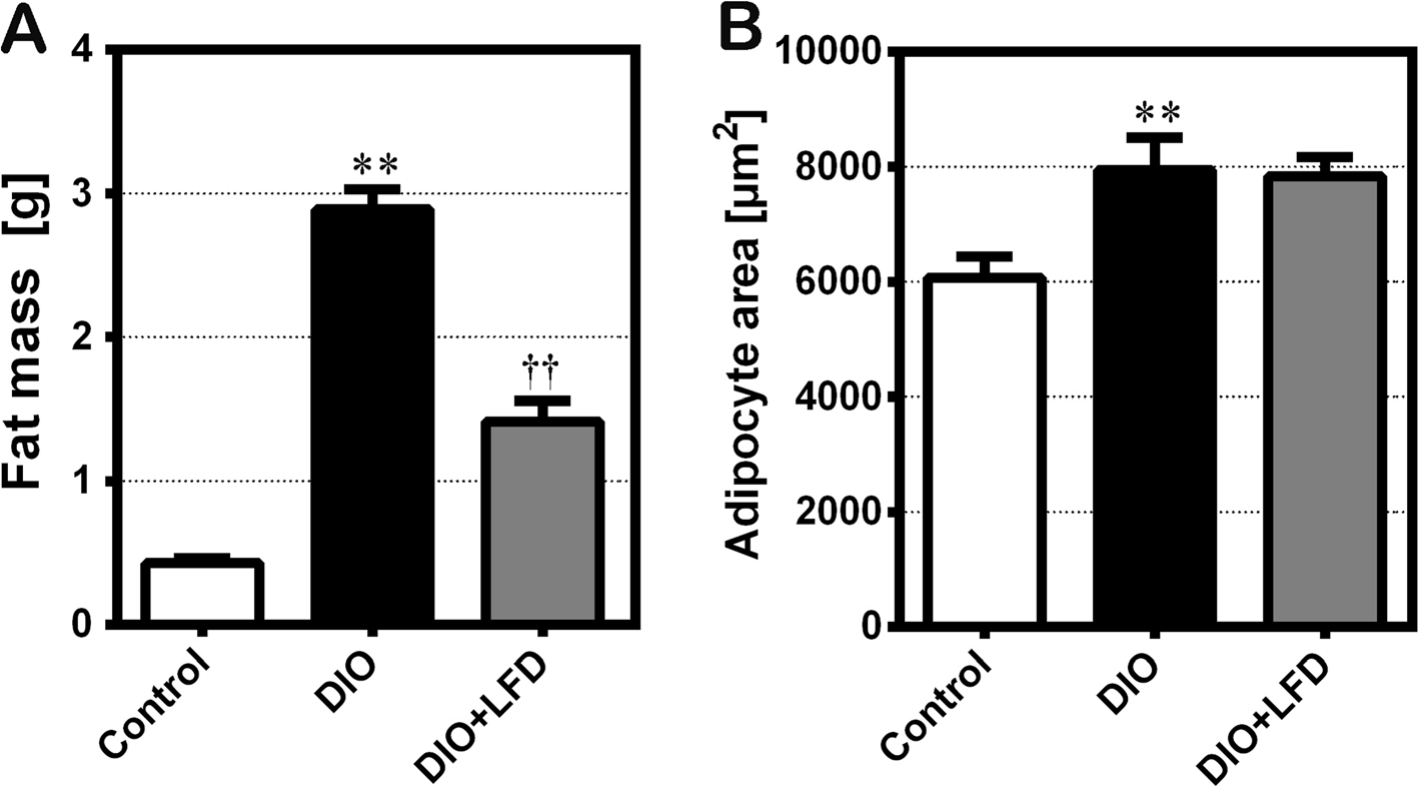
**a** The weight of abdominal mesenteric white adipose tissue and **b** the adipocyte area of C57BL/6NCrl mice in groups of control, DIO, DIO + LFD at 12w. **,*P* < 0.01 vs. control. ††, *P* < 0.01 vs. DIO (*n* = 4 in each group)

**Fig. 3:**
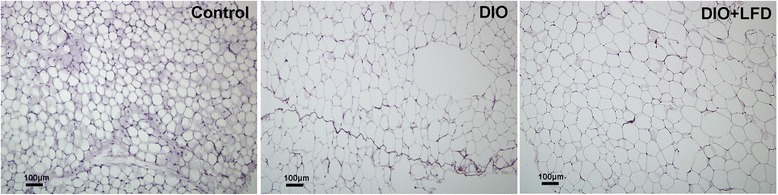
Hematoxylin and eosin stain of the paraffin-embedded white adipose tissue at 5 μm section of C57BL/6NCrl mice in groups of control, DIO, DIO + LFD at 12w

**Fig. 4:**
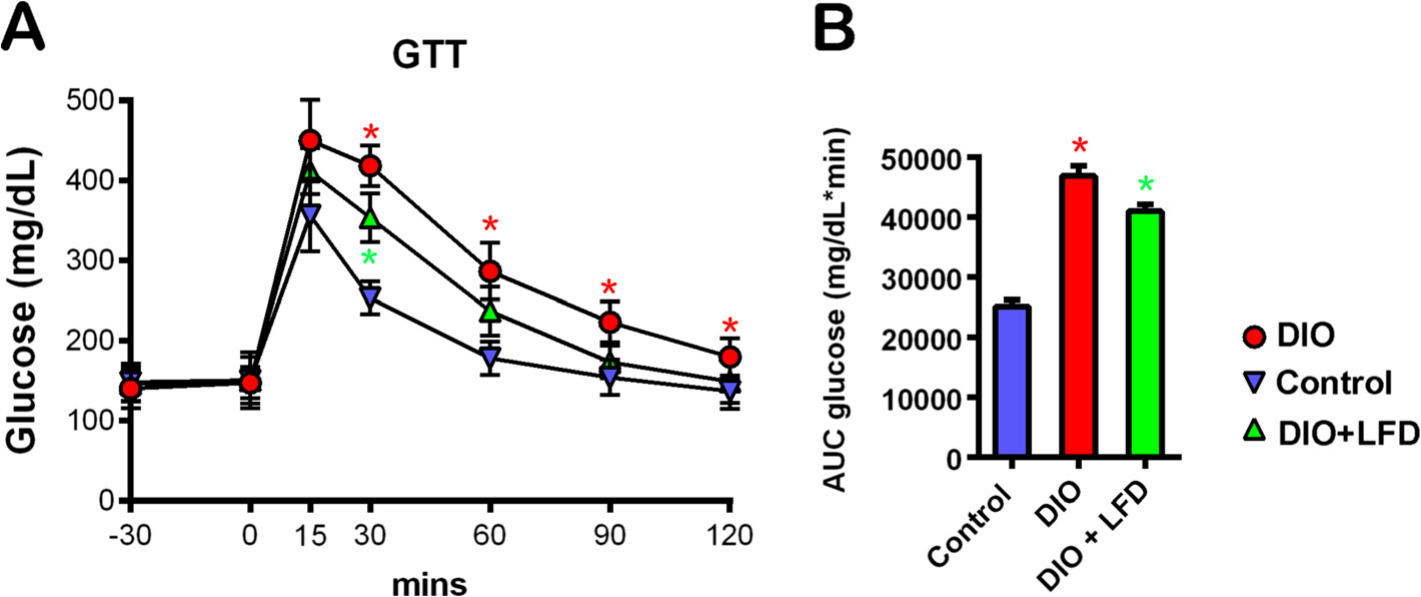
Blood glucose concentrations (**a**) and area under the curve (AUC) quantification (**b**) during a 120-min glucose tolerance test in groups of control, DIO, DIO + LFD at 12w. *(red color), *P* < 0.05 vs. control. *(green color), *P* < 0.05 vs. DIO

In comparison with the C57BL/6NCrl mice fed the standard diet, feeding with the HFD significantly increased body weight (Fig. 1). By contrast, after the change from HFD feeding to LFD feeding, the body weight of DIO mice decreased quickly, with the mean body weight stabilizing after 4 weeks on the LFD. Abdominal mesenteric WAT was also significantly larger in the DIO mice than in control mice, and the mice that were switched to the LFD had significantly less abdominal mesenteric WAT than those that remained on the HFD (Fig. 2a). However, the lower amount of abdominal mesenteric WAT was not accompanied by a significantly smaller adipocyte area (Figs. 2b and 3). Notably, because the fat specimen chosen at 50 μm distance was less than the mean diameter of the adipocytes, there may be exist a selection bias that some measured adipocytes were repeatedly calculated. However, we expect this selection bias is not significant because the tissue section was chosen in a fixed distance and the counting of high number of adipocytes could decrease the deviation of calculated mean adipocyte area. After injection of glucose to the control mice, blood glucose levels increased to a peak of 350 mg/dL after 15 min, and then gradually returned to baseline after 120 min. In the DIO mice, blood glucose concentrations at 30 to 120 min during the GTT were significantly higher than those in the control mice (Fig. 4a). HFD-fed animals displayed significant impairment in glucose tolerance, as evidenced by a 90 % higher incremental glucose AUC (Fig. 4b). In addition, significantly lower glucose level was observed at 30 min after glucose injection for the LFD-fed mice relative to the HFD-fed mice (Fig. 4a), resulting in an around 15 % lower glucose AUC (Fig. 4b).

### LFD did not induce the differential expression of inflammatory cytokines

**Fig. 5:**
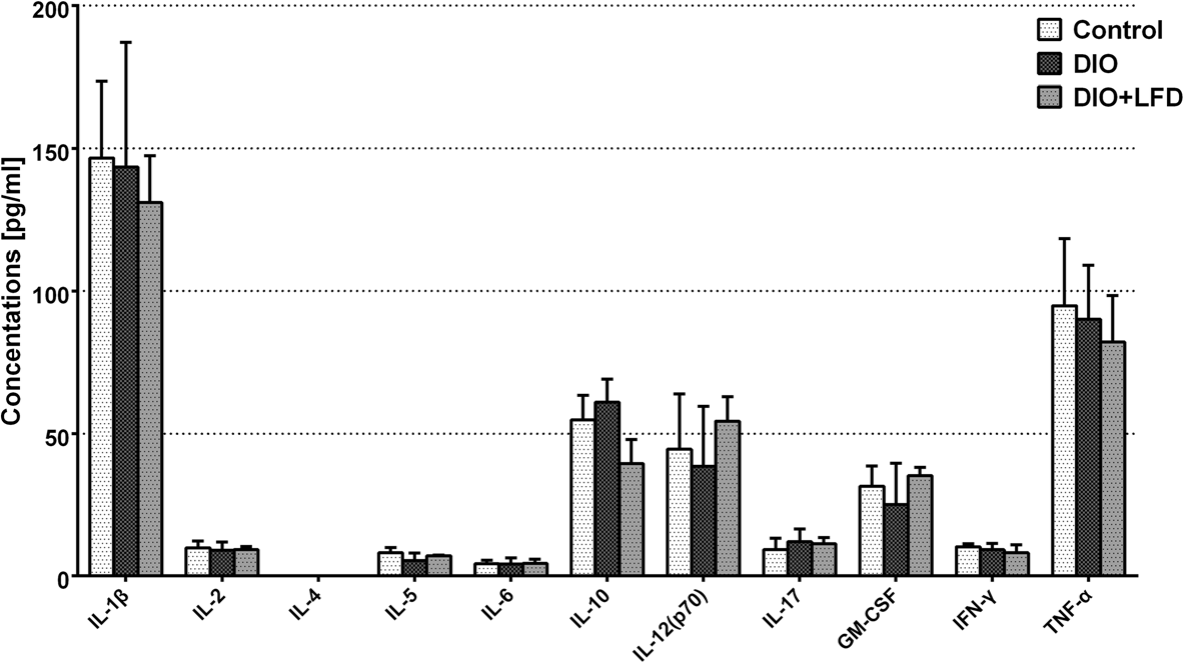
Concentrations of serum cytokines (IL-1β, IL-2, IL-4, IL-5, IL-6, IL-10, IL-12(p70), IL-17, GM-CSF, IFN-γ, TNF-α) analyzed by the Bio-Plex Multiplex cytokine assay at 12w in the mice in groups of control, DIO, DIO + LFD (*n* = 6 in each group)

Of the 11 biomarkers measured (IL-1β, IL-2, IL-4, IL-5, IL-6, IL-10, IL-12 (p70), IL-17, GM-CSF, IFN-γ, and TNF-α), IL-1β had the highest expression in serum, followed by TNF-α, IL-10, and IL-12 (p70) (Fig. 5). However, there were no significant differences in expression among the groups.

### Upregulated miRNA targets in microarray analysis

**Fig. 6:**
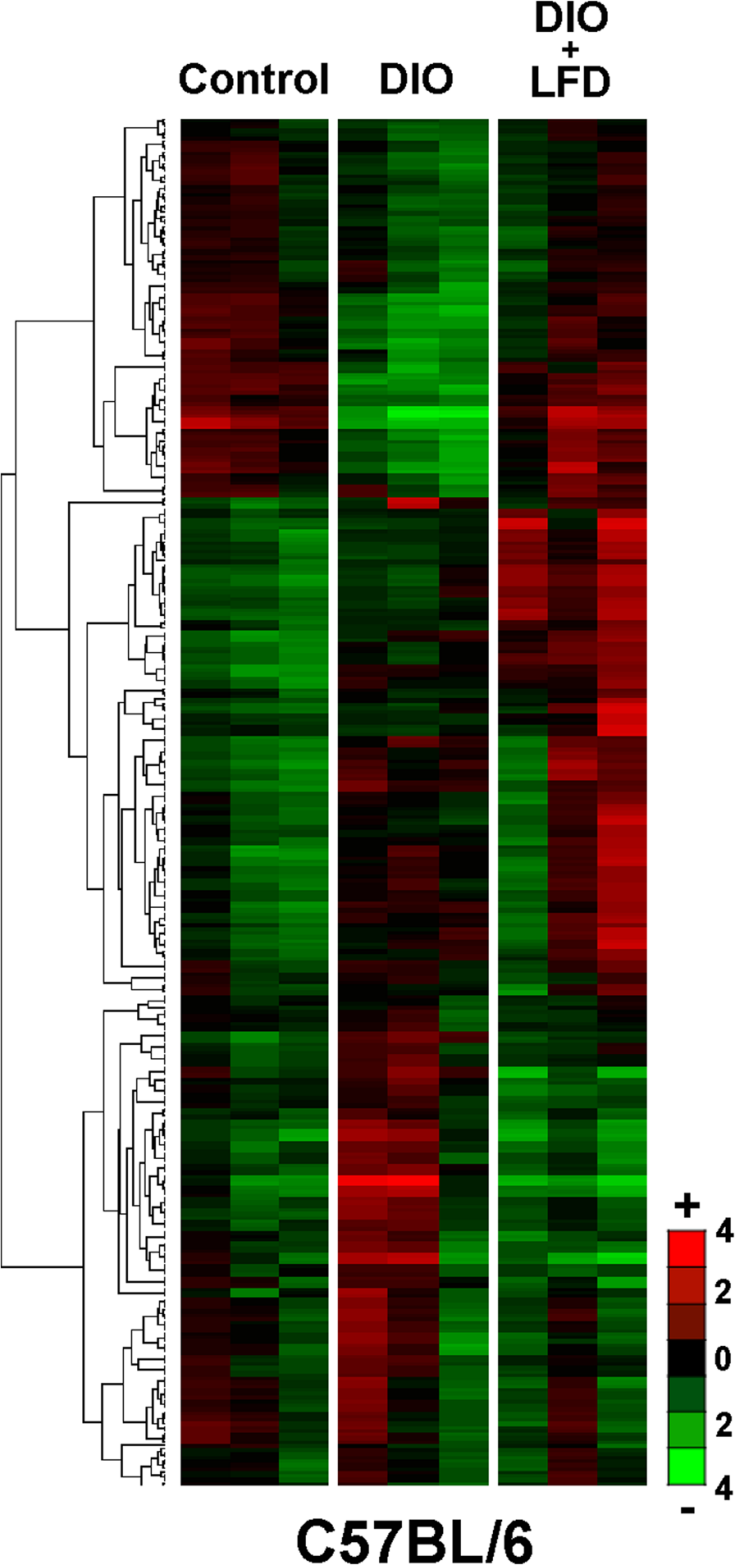
Hierarchical cluster analysis of differentially expressed circulating miRNAs in the serum of C567BL/6 mice in groups of control, DIO, DIO + LFD (*n* = 3 in each group)

**Fig. 7:**
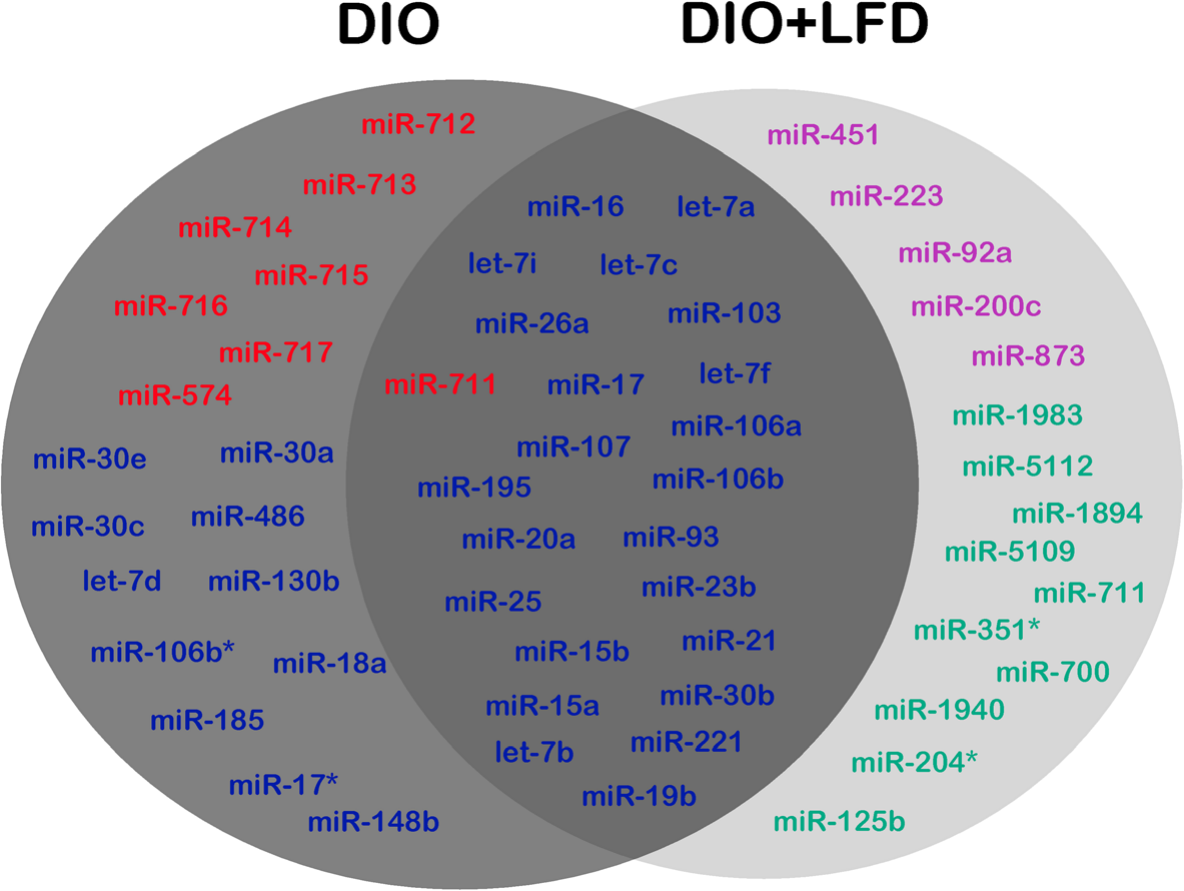
Venn diagram of the changed miRNAs in the serum of C567BL/6 mice in groups of DIO and DIO + LFD

Greater than 2-fold differences in serum miRNA expression between DIO mice and controls as well as between DIO + LFD and DIO mice (*p* < 0.05) were identified for further analysis. The microarray and qPCR results were in agreement, with a Pearson correlation value of 0.891 (Additional file 1), under the limitation of only five miRNAs being selected for analysis. Unsupervised hierarchical clustering of all differentially expressed serum miRNAs was conducted to separate samples from experimental or control subjects into different groups (Fig. 6). In microarray experiments of DIO mouse sera, eight miRNAs were upregulated, and 34 were downregulated (Table 1). In addition, in the sera of DIO + LFD mice, 28 miRNAs were upregulated, and 10 were downregulated (Table 2). As shown in the Venn diagram in Fig. 7, notably, 23 of the 28 upregulated miRNAs in DIO + LFD mice (mmu-miR-16, mmu-let-7i, mmu-miR-26a, mmu-miR-17, mmu-miR-107, mmu-miR-195, mmu-miR-20a, mmu-miR-25, mmu-miR-15b, mmu-miR-15a, mmu-let-7b, mmu-let-7a, mmu-let-7c, mmu-miR-103, mmu-let-7f, mmu-miR-106a, mmu-miR-106b, mmu-miR-93, mmu-miR-23b, mmu-miR-21, mmu-miR-30b, mmu-miR-221, and mmu-miR-19b) were downregulated in the DIO mice. Only five miRNAs (mmu-miR-451, mmu-miR-223, mmu-miR-92a, mmu-miR-200c, and mmu-miR-873) were differentially expressed, implying that the majority of miRNA downregulation associated with obesity could be reversed by LFD treatment. By contrast, of the eight upregulated miRNAs in DIO mice, only one (mmu-miR-711) was significantly downregulated in DIO + LFD mice.

**Table 1:** miRNA targets dys-regulated more than 2-fold in serum of C57BL/6NCrl mice at 12w high-fat diet-induced obesity vs. control (*P*-value < 0.05)

Up-regulated			Down-regulated		
DIO	Fold (log_2_)	*p*-value	DIO	Fold (log_2_)	*p*-value
mmu-miR-711	2.15	0.00	mmu-let-7i	−3.98	0.01
mmu-miR-712	1.58	0.04	mmu-miR-16	−3.83	0.01
mmu-miR-713	1.41	0.03	mmu-miR-15a	−3.02	0.01
mmu-miR-714	1.12	0.05	mmu-miR-26a	−2.61	0.00
mmu-miR-715	1.07	0.01	mmu-miR-107	−2.53	0.00
mmu-miR-716	1.05	0.04	mmu-miR-106b	−2.51	0.01
mmu-miR-717	1.05	0.05	mmu-miR-17	−2.49	0.00
mmu-miR-574	1.03	0.03	mmu-miR-93	−2.36	0.00
			mmu-let-7b	−2.33	0.00
			mmu-miR-15b	−2.31	0.02
			mmu-miR-25	−2.26	0.04
			mmu-let-7c	−2.20	0.00
			mmu-miR-20a	−2.17	0.00
			mmu-miR-103	−2.16	0.00
			mmu-miR-221	−1.97	0.00
			mmu-miR-195	−1.86	0.03
			mmu-miR-21	−1.85	0.00
			mmu-miR-106a	−1.82	0.00
			mmu-let-7a	−1.70	0.01
			mmu-let-7f	−1.67	0.04
			mmu-miR-30c	−1.55	0.05
			mmu-let-7d	−1.52	0.02
			mmu-miR-19b	−1.41	0.01
			mmu-miR-30e	−1.35	0.02
			mmu-miR-30a	−1.34	0.02
			mmu-miR-23b	−1.32	0.03
			mmu-miR-486	−1.28	0.03
			mmu-miR-30b	−1.25	0.01
			mmu-miR-130b	−1.25	0.05
			mmu-miR-106b*	−1.14	0.02
			mmu-miR-185	−1.10	0.03
			mmu-miR-18a	−1.06	0.01
			mmu-miR-17*	−1.04	0.04
			mmu-miR-148b	−1.01	0.04

**Table 2:** miRNA targets dys-regulated more than 2-fold in serum of C57BL/6NCrl mice following 4 w low-fat diet after 8 w high-fat diet-induced obesity vs. 12 w high-fat diet-induced obesity (*P*-value < 0.05)

Up-regulated			Down-regulated		
DIO + LFD	Fold (log_2_)	*p*-value	DIO + LFD	Fold (log_2_)	*p*-value
mmu-miR-16	3.48	0.00	mmu-miR-1983	−3.29	0.05
mmu-let-7i	3.23	0.01	mmu-miR-5112	−2.39	0.02
mmu-miR-26a	2.57	0.02	mmu-miR-1894	−2.18	0.02
mmu-miR-17	2.38	0.02	mmu-miR-5109	−1.88	0.01
mmu-miR-107	2.35	0.02	mmu-miR-711	−1.80	0.00
mmu-miR-451	2.34	0.00	mmu-miR-351*	−1.37	0.04
mmu-miR-195	2.28	0.01	mmu-miR-700	−1.19	0.01
mmu-miR-20a	2.06	0.01	mmu-miR-1940	−1.15	0.03
mmu-miR-25	1.88	0.03	mmu-miR-204*	−1.14	0.02
mmu-miR-15b	1.85	0.01	mmu-miR-125b	−1.09	0.03
mmu-miR-15a	1.83	0.01			
mmu-let-7b	1.73	0.00			
mmu-let-7a	1.69	0.00			
mmu-let-7c	1.64	0.02			
mmu-miR-103	1.61	0.01			
mmu-let-7f	1.57	0.01			
mmu-miR-106a	1.56	0.01			
mmu-miR-106b	1.48	0.04			
mmu-miR-93	1.37	0.01			
mmu-miR-23b	1.34	0.02			
mmu-miR-21	1.23	0.01			
mmu-miR-223	1.18	0.04			
mmu-miR-30b	1.16	0.02			
mmu-miR-221	1.11	0.01			
mmu-miR-19b	1.06	0.02			
mmu-miR-92a	1.05	0.02			
mmu-miR-200c	1.03	0.02			
mmu-miR-873	1.00	0.04

### Target prediction and function annotation

**Fig. 8:**
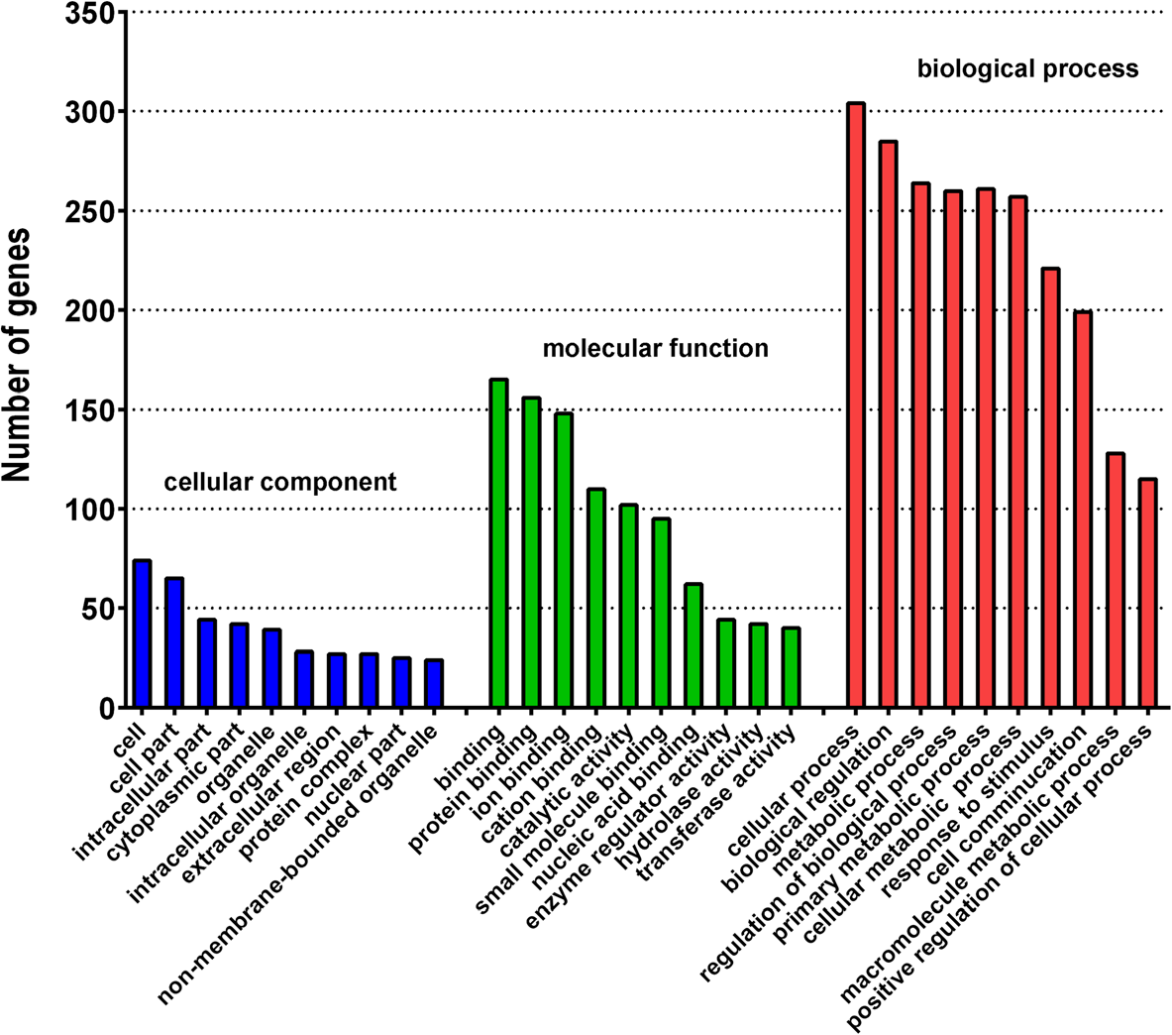
Partial gene ontology (GO) classification annotated for the predicted target genes of 23 interesting up-regulated miRNAs. The figure shows partial GO enrichment for the predicted target genes in cellular component, molecular function, and biological process. All the *P* < 0.001

To further understand the physiological functions and biological processes associated with the 23 miRNAs, target prediction was performed by integrating three public databases (TargetScan, PicTar, and miRanda), and 1082 target genes were identified. GO annotation and KEGG pathway analysis was also performed to identify functional modules regulated by these 23 miRNAs. In GO annotation analysis, cellular processes, biological regulation, metabolic processes, primary metabolic processes, and cellular metabolic processes were the most significantly enriched GO terms (Fig. 8). KEGG pathway analysis revealed 142 pathways associated with these miRNA targets. Among these, metabolic pathways were the most enriched, with 1024 associated genes, followed by MAPK signaling, actin cytoskeleton regulation, secondary metabolite biosynthesis, focal adhesion, insulin signaling, calcium signaling, cytokine-cytokine receptor interaction, tight junctions, phagosomes, and adipocytokine signaling pathways (Table 3). These results suggest that these targets have a high possibility of being regulated by miRNAs during obesity and weight reduction through LFD feeding; however, the possibility of false-positive results from the prediction algorithm always exists.

**Table 3:** The most enriched KEGG pathways of target genes for 23 differentially expressed miRNAs between the sera of C57BL/6NCrl mice during obesity and weight reduction by low-fat diet

Pathway	Count of genes	*P*-value	Pathway ID
Metabolic pathways	1024	2.11E06	ko01100
MAPK signaling pathway	514	1.82E05	ko04010
Regulation of actin cytoskeleton	471	3.09E05	ko04810
Biosynthesis of secondary metabolites	392	4.72E05	ko05200
Focal adhesion	342	6.21E10	ko04510
Insulin signaling pathway	318	5.13E05	ko04910
Calcium signaling pathway	295	5.34E05	ko04020
Cytokine-cytokine receptor interaction	228	2.16E04	ko04060
Tight junction	172	4.22E06	ko04530
Phagosome	144	2.14E05	ko04145
Adipocytokine signaling pathway	117	1.27E04	ko04920

## Discussion

Switching to a LFD is an effective intervention to promote weight loss and improve metabolic health parameters in obesity [19]. Although morbid obesity is considered a systemic inflammatory state, the serum inflammatory profile of C57BL/6 mice, as measured by an antibody array, revealed that DIO mice had higher leptin, IL-6, and LPS-induced chemokine concentrations and lower concentrations of all other chemokines/cytokines than control mice [20]. In this study, we demonstrated that LFD feeding reduced the body weight and adiposity of DIO mice; however, there was no significant difference in the expression of the 11 measured cytokines between DIO and DIO + LFD mice, suggesting that DIO mice may be in an early state of obesity. By contrast, significantly dysregulated miRNAs were identified in both groups. Notably, most (23 of 28) of these circulating miRNAs were upregulated in DIO + LFD and downregulated in DIO mice, implying that the downregulation of these miRNAs by obesity could be reversed by LFD treatment. In addition, target prediction and function annotation revealed that the target genes associated with these 23 differentially expressed miRNAs are involved in metabolic, insulin, and adipocytokine signaling pathways that directly link the pathophysiological changes that occur during obesity and weight reduction. Therefore, whether miRNA supplementation represents a potential therapeutic strategy to treat obesity is an interesting topic requiring further robust investigations to clarify.

As many miRNAs are extensive regulators of adipocyte development and function, their differential expression in the adipose tissue of mice in response to HFD-induced obesity has been explored and discussed in a number of articles [11–13, 21]. For example, in a microarray experiment, 26 miRNAs were upregulated in WAT in response to HFD feeding, including mmu-miR-342-3p, mmu-miR-222, mmu-miR-221, mmu-miR-142-3p, mmu-miR-142-5p, mmu-miR-21, mmu-miR-335-5p, mmu-miR-146a, mmumiR-146b, mmu-miR-647*, and mmu-miR-379, whereas the following miRNAs were downregulated: mmu-miR-141, mmu-miR-200a, mmu-miR-200b, mmumiR-200c, mmu-miR-122, mmu-miR-204, mmu-miR-133b, mmu-miR-1, mmu-miR-30a*, mmu-miR-130a, mmu-miR-192, mmu-miR-193a-3p, mmu-miR-203, mmu-miR-378, and mmumiR-30e* [21]. Some of the circulating miRNAs identified in this study have also been reported in the adipose tissue of DIO mice or implicated in adipogenic processes [11–13], including *Let-7*, miR-103, miR-15, the miR-17-92 cluster (miR-17, miR-20a, and miR-92a), miR-21, miR-221, and miR-30b. However, these miRNA that were previously reported to be upregulated in adipose tissue were downregulated in DIO mice but upregulated in DIO + LFD mice in this study, casting doubt on the suggestion that these circulating miRNAs originated from the adipose tissue of obese mice. Notably, although the origin of circulating miRNAs is debatable, the expression profile of circulating miRNAs is obviously different from those in pathological tissues [22–24]. Whether these circulating miRNAs originated from adipose tissue, blood cells, or other cells of the circulating system requires further experimentation to clarify.

In this study, some of the identified dysregulated miRNAs have been linked to obesity or adipogenesis in the literature. Among them, five members of the Let-7 family (mmu-let-7a, mmu-let-7b, mmu-let-7c, mmu-let-7f, and mmu-let-7i) were dysregulated in response to obesity and weight reduction following LFD feeding. Mice with global overexpression of *Let-7* are viable, but they have reduced body size and weight [25]. In mice, 12 genes encode members of the *Let*-7 family, which includes nine slightly different miRNAs (*Let*-7a, *Let*-c, and *Let*-7f [all encoded by two genes], and *Let*-7b, *Let*-7d, *Let*-7e, *Let*-7g, *Let*-7i, and miR-98 [all encoded by one gene]). All *Let*-7 family members are believed to have similar functions because they share a common seed region (nucleotides 2–8), which mediates interactions between miRNA and target mRNAs [25]. Furthermore, *Let-7* transgenic mice exhibit impaired glucose tolerance because of diminished glucose-induced insulin secretion, and anti-miR–induced silencing of *Let-7* has been proven to improve blood glucose levels and insulin resistance in obese mice [25].

*In vivo*, miR-103 is downregulated in the mature adipocytes of obese mice [26] and upregulated during the differentiation of human and murine pre-adipocytes [27, 28]. A 9-fold upregulation of miR-103 was noted during early adipogenesis in 3T3-L1 pre-adipocyte cells, and lipid droplet formation was accelerated when it was ectopically expressed [26]. miR-103 is also upregulated during porcine adipogenesis, and its inhibition suppresses adipogenesis [26].

miR-15a overexpression leads to a decrease in the number, but an increase in the size, of murine adipocytes by inhibiting Delta-like 1 homolog expression [29]. In a study of miRNA libraries reconstructed from pre- and post-differentiated 3T3-L1 cells, it was noted that miR-15a may not be related to the actual differentiation process, but it may induce growth arrest and/or hormonal stimulation [30]. In addition, the miR-17-19 cluster, which comprises seven miRNAs (miR-17-5p, miR-17-3p, miR-18, miR-19a, miR-20, miR-19b, and miR-92-1) and promotes cell proliferation in various cancers, has been demonstrated to be significantly upregulated at the clonal expansion stage of adipocyte differentiation. MiR-17-92 has been revealed to target Rb2/p130, an important early regulator of pre-adipocyte clonal expansion [31], and the stable transfection of 3T3L1 cells with miR-17-92 resulted in accelerated differentiation and increased triglyceride accumulation after hormonal stimulation [32]. The adipogenic miR-21 has also been demonstrated to be upregulated in human obesity [33] and to enhance adipogenesis in human adipose tissue-derived mesenchymal stem cells (hASCs) by mediating TGF-β signaling [34].

The miR-30 family has been found to be important for adipogenesis [12]. In this study, miR-30a, miR-30b, and miR-30c were significantly downregulated in obese mice, and miR-30b was significantly upregulated after LFD feeding. MiR-30 family members are strongly upregulated during adipogenesis in human cells, and inhibition of miR-30 inhibits adipogenesis [12]. miR-30 family members have also been demonstrated to act as positive regulators of adipocyte differentiation in a human adipose tissue-derived stem cell model [35]. Overexpression of miR-30a and miR-30d stimulates adipogenesis, and it has been demonstrated that miR-30a and miR-30d target RUNX2, a major regulator of osteogenesis and a potent inhibitor of PPARγ, the master gene in adipogenesis [36]. MiR-30c has been found to be upregulated in adipogenesis and to enhance adipogenesis in hASCs, and it appears to target two genes (PAI-1 and ALK2) in distinct pathways [37]. Moreover, miR-30d has been identified as a positive regulator of insulin transcription [38].

Furthermore, in this study, eight miRNAs (mmu-miR-711, mmu-miR-712, mmu-miR-713, mmu-miR-714, mmu-miR-715, mmu-miR-716, mmu-miR-717, and mmu-miR-574) were upregulated in DIO mice. Of these, miR-712 is a mechanosensitive miRNA that is upregulated in endothelial cells by disturbed flow, which regulates endothelial dysfunction and atherosclerosis [39, 40]. MiR-717, which was first reported in mice, is encoded by intron 3 of the body mass-associated glypican-3 (Gpc3) gene, and it plays an important regulatory role in renal osmoregulation. Meanwhile, Gpc3 knockout mice display increased body mass, renal dysplasia, and perinatal mortality [41]. Bioinformatics analysis enables functional annotation of MiR-717 orthologs to determine the effect of its target genes on fat-related traits [42]. However, the effects and mechanisms of these eight upregulated miRNAs in the obese mice in this study are poorly understood.

## Conclusion

This study identified the expression profile of circulating miRNAs in a mouse model of DIO and DIO with subsequent weight reduction through LFD feeding. The results demonstrated that the majority of miRNA downregulation in association with obesity could be reversed by LFD feeding. Target prediction and function annotation revealed that the target genes associated with these 23 differentially expressed miRNAs are involved in metabolic, insulin, and adipocytokine signaling pathways that directly link the pathophysiological changes that occur during obesity and weight reduction.

## Additional file

Additional file 1:
**Correlation of the miRNA expression in the microarray and qPCR.** (TIFF 83 kb).
