# Supplementary figures and images for: Weight-reduction through a low-fat diet causes differential expression of circulating microRNAs in obese C57BL/6 mice
Source: BMC Genomics. 2015 Sep 16;16(1):699. doi: 10.1186/s12864-015-1896-3 (PMC4571067; doi:10.1186/s12864-015-1896-3)

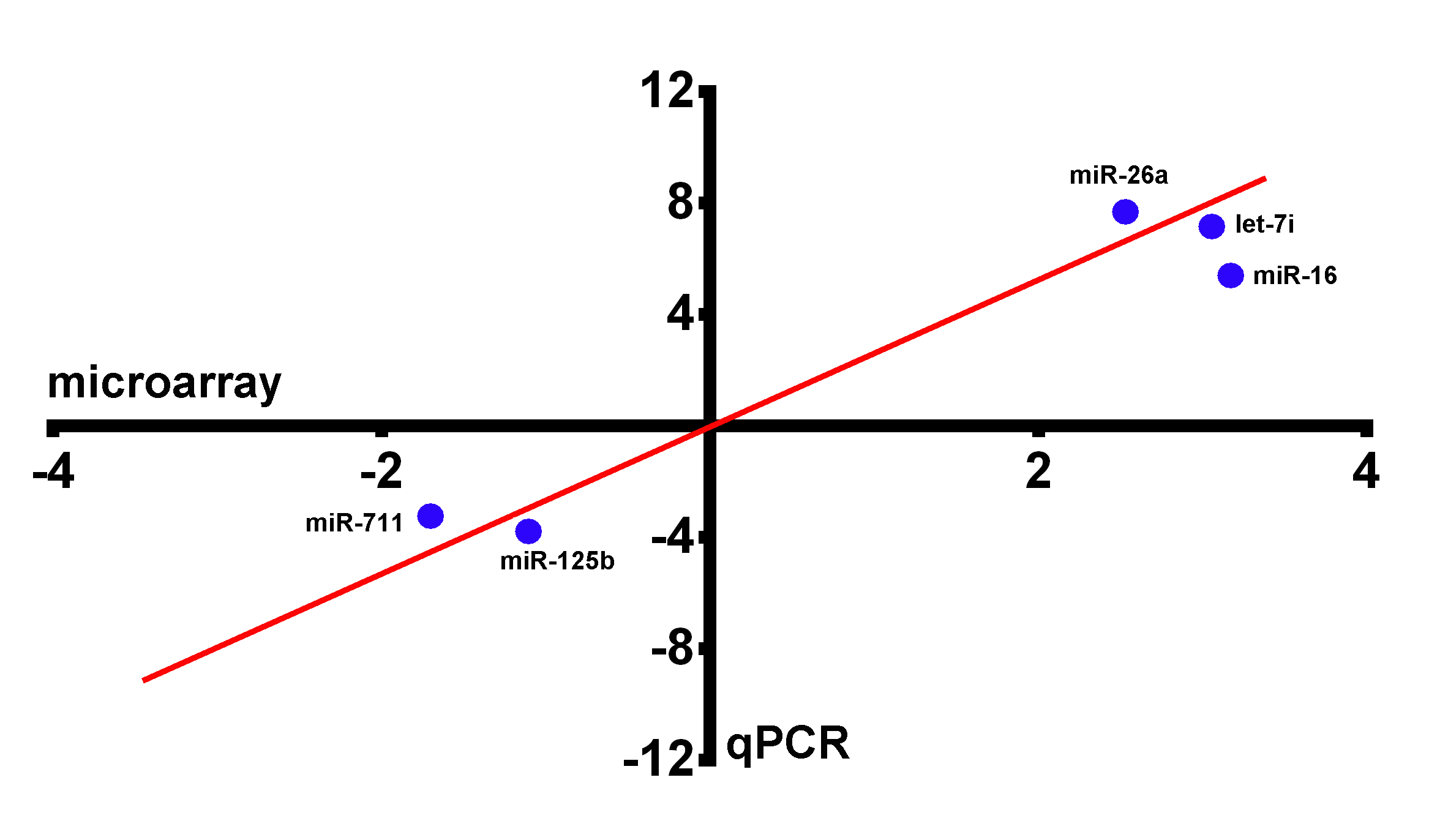

Supplement: Additional file 1: — Correlation of the miRNA expression in the microarray and qPCR. (TIFF 83 kb). [file 12864_2015_1896_MOESM1_ESM.tiff]
